# An efficient harvesting strategy for agarwood based on the correlation analysis of resin formation and leaves dynamic changes induced by integrated induction method

**DOI:** 10.1371/journal.pone.0327516

**Published:** 2025-07-10

**Authors:** Jie Chen, Tianyu Gao, Yanhui Ge, Xiaodong Chen, Meirou Feng, Xiaoying Chen, Weimin Zhang, Xiaoxia Gao

**Affiliations:** 1 School of Pharmacy, Guangdong Pharmaceutical University, Guangzhou, Guangdong, China; 2 Guangzhou Renheng Pharmaceutical, Guangzhou, Guangdong, China; 3 Sirio Pharma Co., Ltd, Shantou, Guangdong, China; 4 Yuebei People’s Hospital, Shaoguan, Guangdong, China; 5 Guangdong Academy of Sciences, State Key Laboratory of Applied Microbiology Southern China, Guangdong Provincial Key Laboratory of Microbial Culture Collection and Application, Institute of Microbiology, Guangzhou, Guangdong, China; Central University of Punjab, INDIA

## Abstract

Agarwood is a resin produced by wounded *Aquilaria* plants. *Aquilaria sinensis* (Lour.) Gilg  is the original plant source of agarwood in China. Formic acid combined with *Botryosphaeria rhodina* A13 (FAA13) induces the formation of artificial agarwood as an effective integrated induction method. However, its formation mechanism is still unclear, and the harvesting time of agarwood has not been elucidated. In this work, we analyzed FAA13-induced artificial agarwood and leaves at different time points within one year based on endophytic fungal community, expression of related genes, and secondary metabolites. The induction process by FAA13 was divided into two stages. In agarwood, we found that fungal diversity and relative abundance decreased in stage 1 but increased in stage 2. Additionally, genes related to 2-(2-phenylethyl) chromones synthesis were mainly expressed in stage 1, while those related to sesquiterpene synthesis were mainly expressed in stage 2. The primary differential metabolites between the two stages were the content of ethanol-soluble extractives (EEC%) in the agarwood and epi-friedelinol and friedelin in the leaves. EEC% in agarwood stabilized and was at a high level in stage 2. At the same time, we observed friedelin rose rapidly from a plateau or after a slight decline, and epi-friedelinol continued to rise. We found similar results in artificial agarwood induced by combining formic acid with *Fusarium* sp. A2 (FAA2). The content of epi-friedelinol and friedelin in leaves can be used as an index to judge agarwood’s harvesting period during the integrated method’s induction process. The appropriate harvesting period for agarwood should be determined by collecting leaves in stage 2 (8 months later) without damaging the tree and assessing whether friedelin enters a rapid rise from the plateau stage by rapidly determining epi- friedlinol and friedelin content.

## Introduction

Agarwood is a highly valuable aromatic dark resinous heartwood [1]. It is not only a unique spice but also a traditional valuable herb which plays an important role in the treatment of cardiovascular and nervous system diseases [2,3]. *Aquilaria sinensis* (Lour.) Gilg (Thymelaceae) is the original plant source of agarwood. Only after being induced by injury will *A. sinensis* secrete resin at the wound, and after months or years of accumulation, the resin-containing xylem is cut out to obtain the agarwood. According to how the resin is formed, agarwood can be categorized into natural and artificial. The types of chemical components of natural and artificial agarwood are the same, dominated by sesquiterpenes, 2-(2-phenylethyl) chromones, aromatics, triterpenes, and fatty acid compounds, the first three of which are the characteristic components of agarwood [4]. The quality of agarwood is mainly evaluated by many indicators, such as the degree of color, water immersion, aroma, oil and grease percentage, the content of ethanol-soluble extractives (EEC%), agarotetrol, 2-(2-phenylethyl) chromones, sesquiterpenes, γ-eudesmol, ledol, and aristolone [5–9]. Among them, the contents of 2-(2-phenylethyl) chromones and sesquiterpenoids are common indicators for the evaluation of agarwood, while EEC% has gained increasing importance in the quality evaluation of agarwood in recent years and is regarded as a key indicator in assessing its quality. Both the Forestry Industry Standard of the People’s Republic of China (LY/T 2094–2017) and the Pharmacopoeia of People’s Republic of China 2020 (ChP2020) stipulate that EEC% of commercial and medicinal agarwood shall not be less than 10.0%. Besides China, some Southeast Asian countries (e.g., Indonesia) also use this classification method. The higher the EEC%, the better the quality of the agarwood [5,9–11].

In natural conditions, natural agarwood is mainly formed when wild *A.* spp. is attacked by lightning, wind, insects, and ants [1]. The natural agarwood formation cycle is long, and its resources have been over-exploited, making its value high. Artificial induction methods are categorized into physical injury, fungal infection, chemical, and integrated induction; each has its pros and cons. Physical injury method, such as drilling and heat shock, are labor-intensive, leading to significant individual variations, unstable quality, and low resin yield efficiency [1]. Fungal infection method is safe, but it is time-consuming and has low agarwood yield efficiency. Some of these fungi are derived from endophytic fungi of *Aquilaria* plants and belong to genera such as *Fusarium*, *Colletotrichum*, *Botryosphaeria*, etc [12–15]. Chemical induction can induce the production of agarwood more quickly. However, residual chemical inducers in agarwood may have certain toxicity, and proper concentration and precise dosage are required to produce high-quality agarwood. These chemicals mainly include H_2_O_2_, methyl jasmonate, salicylic acid, and formic acid, etc [1,16]. The integrated induction method is a combination of several methods with each other. Among them, the integrated method combining physical injury, fungal infection, and chemical induction can maximize the imitation of the formation process of natural agarwood and produce high-yield and high-quality artificial agarwood in a relatively short time [17–19]. The EEC% of agarwood induced by the integrated induction method (5.5% ~ 38.6%) is better than that of physical injury (4.8% ~ 11.7%), chemical stimulation (2.5% ~ 36.1%) and fungal infection (12.3% ~ 24.2%), and the resulting agarwood is closer to natural agarwood [18].

Agarwood resin formation is a complex process involving various defense mechanisms triggered by injury or fungal elicitors [20,21]. During this process, multiple signaling pathways are involved in the formation of agarwood, among which the most important ones include jasmonic acid signaling, heat shock reaction and H_2_O_2_ signaling pathway. Jasmonic acid plays a key role under heat shock conditions, while H_2_O_2_ and NADPH oxidase play important roles under salt stress [20,22]. These signaling pathways activate the defense mechanisms of *A. sinensis* and contribute to the production of secondary metabolites, such as sesquiterpenoids and 2-(2-phenylethyl) chromones. Furthermore, research shows that specific genes play a crucial role in the induction process of agarwood. Genes such As *AsCHS1*, *AsPKS1* and *AsPKS2* are involved in the synthesis of 2- (2-phenethyl) hormones, while *AS-TPS*, *AS-DXS1* and *AS-DXS2* are involved in the synthesis of sesquiterpenoids. These genes help *A. sinensis* synthesize various stress-resistant substances in response to external pressure [23,24]. During the formation of agarwood, the composition of endophytic fungi and endophytic bacteria undergoes significant changes. The role of fungi cannot be ignored. For instance, endophytic bacteria such as *Acidobacteriota*, *Chlamydiae* and *Basidiomycota* are correlated with enhanced levels of essential agarwood fragrances [25]. Meanwhile, the infection of exogenous fungi also plays an important role in the formation of agarwood. Such as the combination of formic acid and *Fusarium sp.* significantly increased the expression of the *CHS* gene and further promoted the production of terpenoids, the generation of which was closely related to the diversity of fungi [26–28]. Furthermore, fungi such as *Phaeoacremonium rubrigenum* promoted the accumulation of sesquiterpenoids in *A. sinensis* by activating the transcription factor-mediated mevaleric acid network [29]. These findings indicate that the formation of agarwood induced by fungi involves a complex multi-pathway synergistic regulatory mechanism.

The formation process of natural agarwood resin usually takes more than 20 years, making it a scarce and slow-growing commodity that cannot meet high market demand [30,31]. The relatively long growth period of natural agarwood can be shortened through artificial induction. Although the prospects are promising, the technology for artificially inducing agarwood still needs to be optimized. One of the bottlenecks in this optimization process is the destructive testing technology. This study employed an efficient comprehensive method for inducing artificial agarwood (FAA13), analyzing the expression levels of sesquiterpene synthase and chalcone synthase genes, secondary metabolite profiles (including EEC%), endophytic fungal dynamics, during the induction process, and leaf secondary metabolite fluctuations, thereby preliminarily elucidating the mechanism of this induction approach. In addition, this study centered on EEC%, an evaluation indicator for agarwood quality, conducted a correlation analysis of multiple indicators in the agarwood production process induced by FAA13, and discovered and established a non-destructive detection technology for determining the harvest period of agarwood. And through another integrated induction method, formic acid with *Fusarium* sp. A2 (FAA2), the applicability of this detection technology was further verified. Under the premise of not damaging the tree body, judging the formation of agarwood and determining the harvest time can maximize the utilization of the forestry resources of the *A. sinensis* tree, providing a scientific basis for the application of comprehensive induction method to induce the formation of agarwood and improve the sustainable production of agarwood.

## Materials and methods

### Preparation of plant materials

Resinous heartwood and leaves of *A. sinensis* were grown in Xinyi, Guangdong, China (51°49′ N, 19°53′ E) and harvested every two months from September 2012 to July 2013. The plant was taxonomically authenticated by Prof. Yan Hanjing (Department of Medicinal Plants, School of Traditional Chinese Medicine, Guangdong Pharmaceutical University, China).

**Fig 1:**
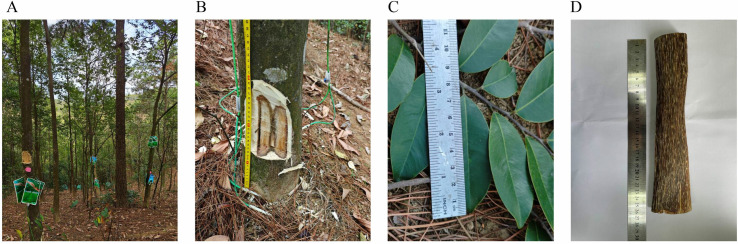
Artificial agarwood induction process and sample diagram. (A) Cultivated agarwood (*A. sinensis*) plantation; (B) Agarwood sampling location inside surface; (C) *A. sinensis* leaves; (D) Agarwood.

Six-year-old *A*. *sinensis* trees, with a height of 3–4 m and a diameter of >10 cm, were used in the experiment. The distance between trees was 50–70 cm. A drill device was used to create holes with a width of 0.5 cm and a depth of 4–5 cm in the tree trunks. Formic acid (1%, analytical grade) was purchased from the Guangzhou chemical reagent factory. *Botryosphaeria rhodina* A13 (EU781670) and *Fusarium* sp. A2 (EU781659) were provided and identified by Prof. Li Haohua (State Key Laboratory of Applied Microbiology Southern China, Guangdong Provincial Key Laboratory of Microbial Culture Collection and Application, Institute of Microbiology, Guangdong Academy of Sciences, Guangzhou). The liquid fungal fermentation product was injected slowly into the xylem of each tree to stimulate resin production. The induction process of the *A. sinensis* and the collection of some samples are shown in Fig 1.

Studies on the inducer of FAA13 were conducted using twenty-one *Aquilaria* trees, divided into 7 groups, with 3 trees in each group. One group consisted of healthy, non-artificially induced *Aquilaria* trees, serving as the control group. The remaining six experimental groups were categorized based on different sampling time points: 2, 4, 6, 8, 10, and 12 months. Samples were not collected from the same tree at consecutive time points to avoid physical damage during artificial collection affecting the resin formation, as continuous physical damage would also lead to resin formation. The leaves and resinous heartwood of *A. sinensis* were harvested at 2, 4, 6, 8, 10 and 12 months after induction, respectively. Resinous heartwood was used for qualitatively analyze the composition of endophytic fungal communities and secondary metabolite profiles, quantitatively determine gene expression levels and EEC%, and leaves were utilized for secondary metabolite profiling and quantitative determination of epi-friedelinol and friedelin.

In addition, the FAA2 inducer was also designed using the same method. Besides 3 trees as the control group, a total of 18 trees were induced and divided into 6 experimental groups according to 6 collection time points. EEC% was determined in FAA2-induced resinous heartwood, while epi-friedelinol and friedelin contents were quantified in leaves.

### Endophytic fungal communities and gene expression analysis

Genomic DNA was extracted from the same amount of resinous heartwood induced by FAA13 using the EZNA soil DNA kit and amplified separately using the fungal primer pair. The amplicons were amplified using TransGen AP221−02: TransStart Fastpfu DNA polymerase and performed in an ABI GeneAmp 9700 (ABI, Carlsbad, USA). A mixture of the amplicons was purified using the AxyPrepDNA gel extraction kit and quantified using QuantiFlour™-ST system (Promega, USA). The mixture was used for pyrosequencing on a Roche 454 GS FLX+ Titanium platform. Sequences that had a length shorter than 200 bp, low quality (average quality score below 25), and ambiguous base calls, as well as those with incorrect primer sequences, were removed. The remaining sequences were assembled into operational taxonomic units (OTUs) using a 97% identity threshold as a criterion. The most abundant sequence from each OTU was selected as a typical sequence for the respective OTU for clustering and classification and against the UNITE database for analysis. Finally, rarefaction analysis and Good’s coverage estimation are carried out.

qRT-PCR assays were conducted using an ABI 7500 real-time PCR system (Applied Biosystems, Foster City, CA) as described previously to investigate the expression of candidate genes from each agarwood sample [27]. The PCR reaction was performed using a THUNDERBIRD SYBR qRT-PCR Mix (Toyobo, Osaka, Japan) and primers, which can be found shown in Table 1. All experiments were repeated at least three times.

**Table 1:** Primers in this study.

Gene	Primer	GenBank Accession Number	Size/bp
*GAPDH*	F-CTGGTATGGCATTCCGTGTA R-AACCACATCCTCTTCGGTGTA	XM003523083	161
*As*-*TPS*	F-TGCAAGAAGATGAAGGAGATGATTG R-GATCACAACCGACGAAGAGATTTC	JQ712683	93
*As-DXS*1	F-AAAGAGGGAGCGTGTTGTAACC R-CACCATATTGCAGTCCAAGTAGCC	JX860325	103
*As-DXS*2	F-GGGCTCTATTACATCGGTCCTG R-CCTTTCTCAGTGACAACGTGGAT	JX860326	122
*As-HMGR*	F-TTCCTACAGAATGATTTCCCTGAC R-TTCTTCACCACATCACCCTTGA	JQ990217	149
*As-CHS*1	F-TCACCAGGAGCGATCACAT R-GGCGACCAGTAGTCAGCAAT	EF103196	139
*As-CHS*2	F-CCAACAGCGAGCACATGACC R-TTCTTTGCCCAACTTCGGGATC	EF103197	194

### Determination of secondary metabolite profile and content by GC-MS

Resinous heartwood and leaves of *A. sinensis* induced by FAA13 were dried at room temperature and then ground into a fine powder (0.2 g) using a pulveriser and passed through No.4 sieve (65 mesh). The powder was then extracted with 10 mL of chloroform (analytical grade; Guangzhou chemical reagent factory) for a 24-hour soak at room temperature. Any lost solution was compensated by adding more chloroform. The extract was filtered through a 0.22 μm microporous membrane to obtain the test solution. Additionally, an alkane retention index standard (C8-C40, 500 mg·L^-1^) was run under the same conditions.

Resinous heartwood was analyzed by GC-MS (Shimadzu QP 2010 Ultra, HP-5 column: 30 m × 0.25 mm × 0.25 μm). The temperatures program was initiated at 90 °C, held for 4 min, then rose at 2.5 °C·min^-1^ to 130 °C, held for 20 min, and then rose at 0.5 °C·min^-1^, to 180°C, held for 5 min then rose at 2.0 °C·min^-1^ to 200 °C. And last rose at 1 °C·min^-1^ to 230 °C, held for 120 min. Helium was used as the carrier gas (1 mL·min^-1^). The MS was operated at an energy of 70 eV, an electronic source temperature of 230 °C, and an interface temperature of 280 °C. The samples were scanned from *m*/*z* 45 to *m*/*z* 500, and the solvent delay was set to 5 min. The sample injection volume was 1 μL, and the injection was operated in splitless mode [32,33].

Leaf samples were analyzed by GC-MS (Agilent 7890A-5975C, HP-5 column: 30 m × 0.25 mm × 0.25 μm). Temperatures program initiated at 130 °C, rose at 30 °C·min^-1^ to 200 °C and then rose at 1 °C·min^-1^ to 280 °C, held for 40 min. The injector temperature was set at 280 °C, and helium was used as the carrier gas (1 mL·min^-1^). The MS was operated at the energy of 70 eV, an electronic source temperature of 230 °C, and an interface temperature of 280°C. The samples were scanned from *m*/*z* 45 to *m*/*z* 500, and the solvent delay was 3 min. The sample injection volume was 0.4 μL, and the injection was set to splitless mode [34,35].

Total ion chromatograms are processed by an Automated Mass Spectrometric Deconvolution Qualitative System (AMDIS). Each common mass peak in the mass spectrum was compared to that in the mass spectrum from the NIST17 standard spectral library, and the retention index RI was used as a criterion for similarity.

### Determination of ethanol-soluble extractives content

The EEC% of agarwood were analyzed using a hot-dip method (General Rules 2201), according to the alcohol-soluble extract content assay in ChP2020 [5]. Take about 2.0 g of resinous heartwood of FAA13 and FAA2 at different time points respectively, weigh accurately, transfer into 100 mL conical flasks, precisely add 50 ml of ethanol, stopper tightly, weigh, stand for 1 h, then connect the reflux condenser, heat to boiling, and maintain a slight boiling for 1 h. After cooling, remove the conical flask, stopper tightly, reweigh. Replenish any weight loss with ethanol dropwise to restore the original weight. Shake well, filter through a desiccator, accurately measure 25 mL of the filtrate, transfer into an evaporating dish that has been dried to a constant weight, evaporate to dryness on a water bath, dry at 105 °C for 3 h, cool in a desiccator for 30 min, and quickly and accurately weigh. Calculate the content (%, w/w) of extractives in the test sample based on the dried product.

### Determination of epi-friedelinol and friedelin content

Leaf samples of FAA13 and FAA2 at different time points were air-dried, crushed and passed through No. 4 sieve (65 mesh), respectively. Take approximately 0.2 g of the prepared powder, weigh accurately, transfer into a glass-stoppered conical flask, precisely add 10 mL of chloroform, stopper tightly, shake well, weigh, and cold soak for 24 h. Reweigh, replenish with chloroform dropwise to original weight, shake well, filter, take the filtrate and filter it through a 0.22 µm microporous membrane to obtain the test solution.

GC-MS analysis of leaves of *A. sinensis* was carried out using Shimadzu GC-MS-QP 2010 Ultra equipped with an HP-5 capillary column (30 m × 0.25 mm × 0.25 μm). The temperatures program was initiated at 130 °C, rose at 30 °C·min^-1^ to 300 °C, and held for 10 min. The injector temperature was 280 °C, and helium was used as the carrier gas (1.5 mL·min^-1^). The MS was operated at 70 eV, and an electronic source temperature of and interface temperature of 230 °C and 280 °C, respectively. The scanning range was between *m*/*z* 50 and *m*/*z* 500, and the solvent delay was 6 min. The sample injection volume was 1 μL, and the injection was in a 1:10 split mode. Sample monitoring was carried out in a selected ion monitoring (SIM) mode.

In process validation, several parameters were evaluated, such as linearity, selectivity, accuracy, precision, and stability, according to General Chapter 9101 of ChP2020. All results were expressed as percentages. The statistic program was used for statistical analysis, and a 5% level of significance was selected.

Two calibration curves were constructed, using epi-friedelinol (a purity of ≥98%, Sichuan Weikeqi Biotechnology Co., LTD.) and friedelin (a purity of ≥98%, Shanghai Yiji Industrial Co., LTD.) at five different concentrations: 1.8–89.2 μg·mL^-1^ for epi-friedelinol, and 1.6–77.6 μg·mL^-1^ for friedelin. Slopes and other statistical parameters of the calibration curves were determined by linear regression. The concentration of samples was fixed at 20.0 mg·mL^-1^, and each sample was prepared in sextuplicate.

Repeatability was assessed from the amount of friedelin and epi-friedelinol obtained at each concentration. The relative standard deviation (RSD) was calculated and used to indicate repeatability.

Recovery was evaluated by the standard addition method, in which a mixture of friedelin and epi-friedelinol with known concentrations was added to the sample extract solution. The concentrations were prepared independently in six replicates. The recovery data were determined by dividing the value for the sample prepared with added standards minus the sample content by the amount added and then multiplying by 100%. The sample solution was stored at room temperature for 36 h and analyzed by GC-MS. The peak area with an RSD of no more than 10% was considered stable.

### Statistical analysis

SPSS and Origin analyzed the correlations between key indicators (month, OUT, gene expression level, EEC% and the content of secondary metabolites). The key indicators data detected in each sample at each of the seven time points were imported as variables into SIMCA14.1 software. Chemical pattern recognition was used to find the differential indicators in the samples at different stages.

## Result

### Dynamic changes of endophytic fungi of FAA13-induced artificial agarwood

To determine the dynamic changes in the endophytic fungal community of FAA13-induced artificial agarwood, we collected samples at various time points: 0, 2, 4, 6, 8, 10, and 12 months. After removing sequences from *Viridiplantae* and other non-fungal sequences, a total of 90522 quality sequences obtained from 102690 reads were classified as fungi. The average length of the quality sequences was 487 bp. Table 2 tabulates the number of OTUs, coverage, and statistical estimates of species richness from each sample with 99% similarity.

**Table 2:** Estimation of phylotypic coverage and diversity of resinous heartwood of *A. sinensis* based on the internal transcribed spacer (ITS) libraries and pyrosequencing analysis.

Month	ACE	Chao	Shannon	Simpson	Coverage
0	196	165	0.56	0.8365	0.9970
2	320	257	1.75	0.3855	0.9952
4	301	197	0.82	0.7014	0.9960
6	72	65	0.53	0.8285	0.9977
8	243	163	0.68	0.798	0.9968
10	131	137	0.63	0.8043	0.9977
12	249	211	0.73	0.7724	0.9956

ACE: The species richness of the sample (A higher ACE value indicates a greater variety of species in the sample).

Chao: Estimate the total number of species in the sample (>50, the sample has a relatively high species diversity).

Shannon: It is usually between 0 and approximately 5. The higher the value, the richer the species diversity in the sample.

Simpson: The range is usually between 0 and 1. The closer the value is to 1, the more uneven the species distribution in the sample.

Coverage: The proportion of species that have been observed in the sample (when close to 1, it indicates that the sample has well represented the overall species diversity).

**Fig 2:**
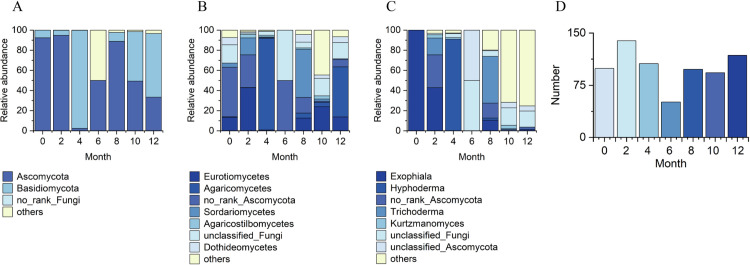
Dynamic changes of fungal diversity of endophytes in FAA13-induced artificial agarwood at different induction times within one year. (A) phylum, (B) class, (C) genus levels, and (D) microfloral abundance of endophytic fungi based on OTU.

The diversity of the endophytic fungal community of *A. sinensis* changed slightly as the FAA13 induction time was increased (Fig 2A). At the phylum level, *Ascomycota* was the dominant community in samples induced for 0 and 2 months, with relative abundances of 73.9% and 93.3%, respectively. However, *Basidiomycota* was dominant in the samples induced for 4 months, with a relative abundance of 93.7%. As the induction duration was prolonged, the relative abundance of *Ascomycota* gradually increased, with relative abundances of 50% and 84.5% in samples induced for 6 and 8 months, respectively. Similar to the results from 0–4 months, after *Ascomycota* became the dominant community, its relative abundance decreased in the samples induced for 10 and 12 months. Similarly, *Basidiomycota* once again dominated the samples induced for 12 months, with a relative abundance of 54.0%.

At the class level, the diversity of endophytic fungal significantly increased after 2 months of FAA13 induction compared to that after 0 months of induction (Fig 2B). After 4–6 months of induction, the diversity of endophytic fungi gradually decreased, and the species of endophytic fungi were gradually becoming single. Although endophytic fungal diversity gradually recovered after 8–12 months of induction, it was still different from that observed in samples induced for 0 months, especially at the genus level (Fig 2C). Seven new genera were discovered after FAA13 induction, including *Cladophialophora*, *Hohenbuehelia*, *Hyphoderma*, *Jattaea*, *Kurtzmanomyces*, *Ocultifur*, and *Phanerochaete*.

### Dynamic changes of relative expression of candidate genes in the tissue of FAA13-induced artificial agarwood

**Fig 3:**
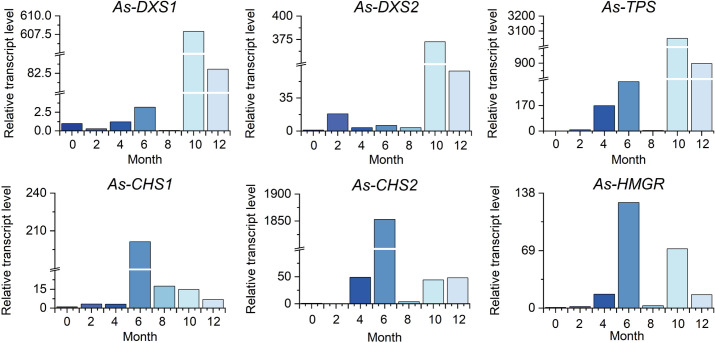
Dynamic changes of expression levels of genes in FAA13-induced artificial agarwood within one year.

To investigate the dynamic changes of relative gene expression in the tissue of artificial agarwood induced by FAA13, the levels of key enzyme genes involved in sesquiterpenes and 2-(2-phenylethyl) chromones biosynthesis were analyzed using qPCR technology. *As-TPS*, *As-DXS1*, and *As-DXS2* genes encode enzymes related to sesquiterpene synthesis. *As-CHS1* and *As-CHS2* encode enzymes related to 2-(2-phenylethyl) chromones synthesis. *HMGR* is involved in the synthesis of both sesquiterpenes and 2-(2-phenylethyl) chromones. As illustrated in Fig 3, the relative expression levels of these genes significantly increased after FAA13 induction. With the 0 months as the blank control group, the relative expression levels of genes were calculated using the 2^-ΔΔ*C*^ method. After induction, the relative expression levels of *As-TPS*, *As-DXS1* and *As-DXS2* genes all increased to varying degrees. Especially in the 10th month of induction, they increased by 3051, 608 and 373 times respectively, and still maintained high expression after 12 months. Similarly, the relative expression levels of *As-HMGR*, *As-CHS1* and *As-CHS2* genes also increased sharply, especially in the 6th month of induction, when the expression levels were the highest, increasing by 127, 201 and 1853 times respectively.

### Dynamic changes of secondary metabolites of artificial agarwood and leaves

**Fig 4:**
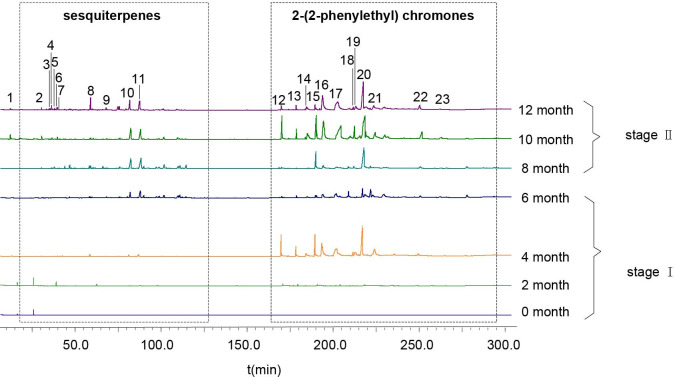
GC-MS total ion chromatograms of agarwood induced by FAA13 (stage Ⅰ: 0−6 months, stage Ⅱ: 8−12 months). 1: benzyl acetone; 2: Isoaromadendrene epoxide; 3 γ-Eudesmol; 4: Guaiol; 5: Diepi-α-cedrene epoxide; 6: Aristolone; 7: Santalol; 8: Baimuxinal; 9: Longifolenaldehyde; 10: Velleral; 11: Verrucarol; 12: 2-(2-Phenylethyl) chromone; 13: 2-[2-(4-hydroxyphenyl) ethyl] chromone; 14: 6-Hydroxy-2-(2-phenylethyl)chromone; 15: 6-Methoxy-2-(2-phenylethyl) chromone; 16: 2-[2-Hydroxy-2-(4-hydroxyphenyl) ethyl] chromone; 17: 8-Hydroxy-2-(2-phenylethyl) chromone; 18: 6-Methoxy-2-[2-(3’-methoxyphenyl) ethyl] chromone; 19: Squalene; 20: 6,7-Dimethoxy-2-(2-phenylethyl) chromone; 21: 6-Hydroxy-7-methoxy-2-(2-phenylethyl) chromone; 22: 6,7-Dimethoxy-2-[2-(4’-methoxyphenethyl)] chromone; 23: 6,8-Dihydroxy-2-[2-(3’-methoxy-4’-hydroxylphenylethyl)] chromone.

**Fig 5:**
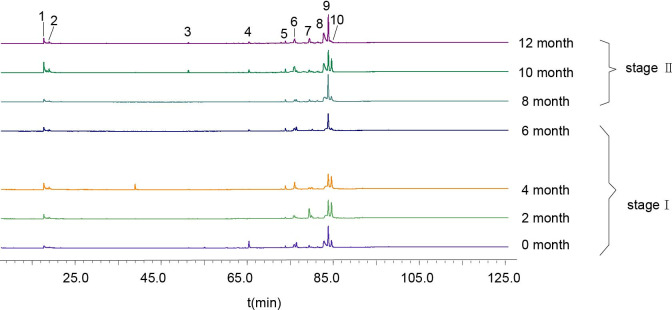
GC-MS total ion chromatograms of leaves induced by FAA13 (stage Ⅰ: 0−6 months, stage Ⅱ: 8−12 months). 1: 3,7,11,15-Tetramethyl-2-hexadecen-1-ol; 2: Phytol; 3: Squalene; 4: Heptacosane; 5: Olean-12-ene; 6: Lupeol; 7: 24-Methylenecycloartan-3-one; 8: Epi-friedelinol; 9: Friedelin; 10: Stigmasterol.

**Fig 6:**
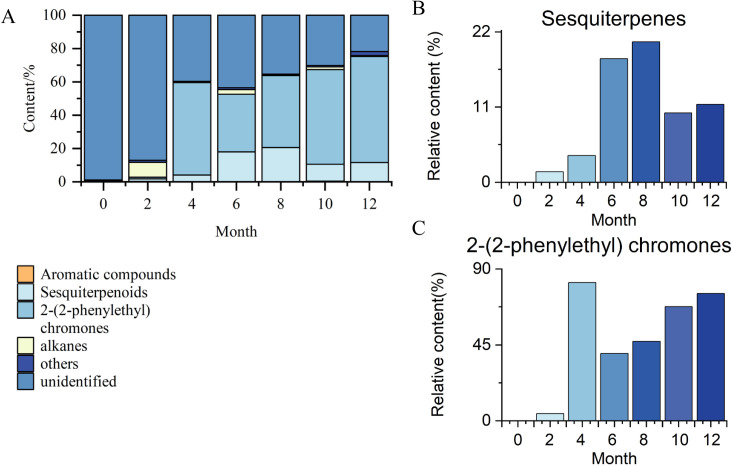
Analysis of GC-MS secondary metabolites of agarwood induced by FAA13. (A) Dynamic changes of secondary metabolites in agarwood. (B) Relative abundance of sesquiterpenes of agarwood. (C) Relative abundance of 2-(2-phenylethyl)chromones of agarwood.

**Fig 7:**
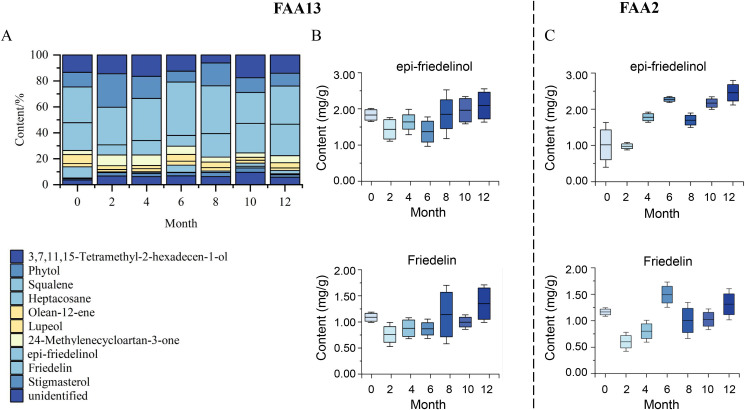
Analysis of GC-MS secondary metabolites of leaves of *A. sinensis.* (A) Dynamic changes of secondary metabolites in leaves of *A. sinensis* induced by FAA13. (B) Content of epi-friedelinol and friedelin in leaves of *A. sinensis* induced by FAA13. (C) Content of epi-friedelinol and friedelin in leaves of *A. sinensis* induced by FAA2.

**Fig 8:**
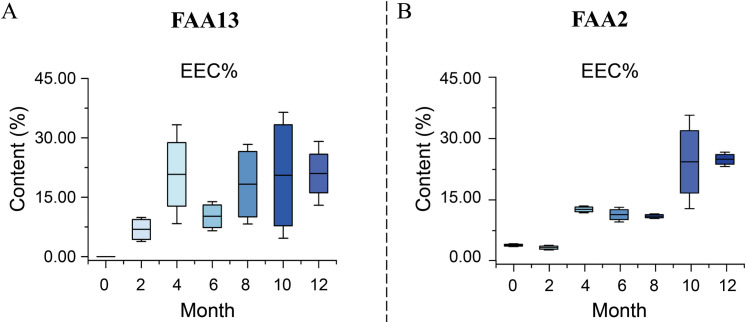
Dynamic changes of EEC% in agarwood within one year. (A) FAA13-induced artificial agarwood. (B) FAA2-induced artificial agarwood.

**Fig 9:**
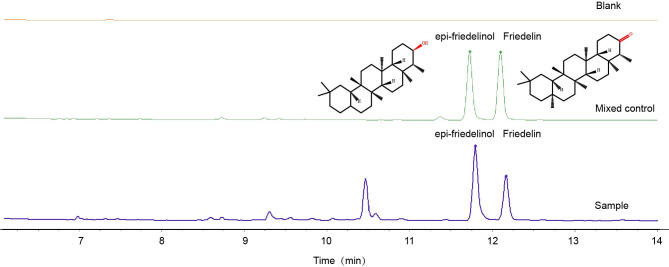
Chromatogram of epi-friedelinol and friedelin isolated from leaves of *A. sinensis* by GC-MS/SIM.

The dynamic changes of secondary metabolites of artificial agarwood and leaves induced by FAA13 analyzed using GC-MS are depicted in Figs 4 and 5. The total ion chromatogram of the samples was processed with AMDIS software, and the extracted mass spectral information was identified by the NIST 17 standard spectral library mass spectral information comparison and RI value correction. The average relative percentage content of each compound was calculated using the GC-MS Postrun Analysis workstation by the area normalisation method. As shown in Fig 6A, sesquiterpenes and 2-(2-phenylethyl) chromones secondary metabolites were detected in FAA13-induced agarwood from the second month. A total of 28 sesquiterpenoids and 16 2-(2-phenylethyl) chromones compositions were detected within 2−12 months, including verrucarol, velleral, baimuxinal, 6,7-dimethoxy-2-(2-phenylethyl) chromone, 6-methoxy-2-(2-phenylethyl) chromone, and 2-(2-Phenylethyl) chromone. The relative contents of total sesquiterpenes in agarwood induced by FAA13 for 2−12 months were 1.443%, 3.883%, 18.067%, 20.520%, 10.159%, and 11.406%, respectively, and the relative contents of total 2-(2-phenylethyl) chromones were 0.920%, 55.551%, 34.468%, 43.159%, 56.709%, and 63.674%, respectively (Fig 6B, C). It is notable that the two types of compounds were not detected until the second month. With increasing induction time, the relative contents of sesquiterpenes and 2-(2-phenylethyl) chromones (S + C) showed an upward trend. The relative contents of total organic acids and esters in agarwood induced by FAA13 for 4−12 months were 5.050%, 2.884%, 4.880%, 1.510%, and 3.863%, respectively. It is notable that these levels were not detected until the fourth month. In the leaves of *A. sinensis* induced by FAA13, 10 compositions were detected, mainly including friedelin, epi-friedelinol, and stigmasterol. As illustrated in Fig 7A, the composition of each sample varied with induction time. The relative content of epi-friedelinol initially decreased but then increased with induction time, while the relative content of friedelin exhibited an opposite trend of increasing and then decreasing. The change of agarwood EEC% over time is shown in Fig 8A. Notably, significant differences were observed in EEC% across different months (*p* = 0.020). EEC% gradually increased from 0 to 4 months of induction but decreased between 4 and 6 months. However, from 6 to 12 months of induction, EEC% exhibited another upward trend. To identify the main differential components in agarwood leaves during the process of FAA13-induced agarwood formation, we developed a method for determining epi-friedelinol and friedelin (Fig 9) in the leaves of *A. sinensis* using GC-MS (SIM) method. The proposed method was validated using established procedures described in materials and methods. The linearity of the curves was within an excellent range (r = 0.9990–0.9994), and the recoveries of epi-friedelinol and friedelin at five concentrations (1.8–89.2 μg·mL^-1^ of epi-friedelinol and 1.6–77.6 μg·mL^-1^ of friedelin) were between 99.3%−104.1% and 99.6% and 105.4%, respectively. Repeatability test revealed that the epi-friedelinol concentration was 1.99 mg·g^-1^ (RSD = 1.0%), and the friedelin concentration was 1.03 mg·g^-1^ (RSD = 1.4%). When the sample solution was kept at room temperature for 24 h, both epi-friedelinol and friedelin had RSDs that were less than 2%, indicating that the sample solutions were stable at room temperature for 24 h. The epi-friedelinol and friedelin contents in the leaves of *A. sinensis* are presented in Fig 7B. The epi-friedelinol and friedelin contents in each month were not significantly different. After induction, the total content of epi-friedelinol and friedelin initially decreased but gradually increased thereafter.

### Correlation analysis

**Fig 10:**
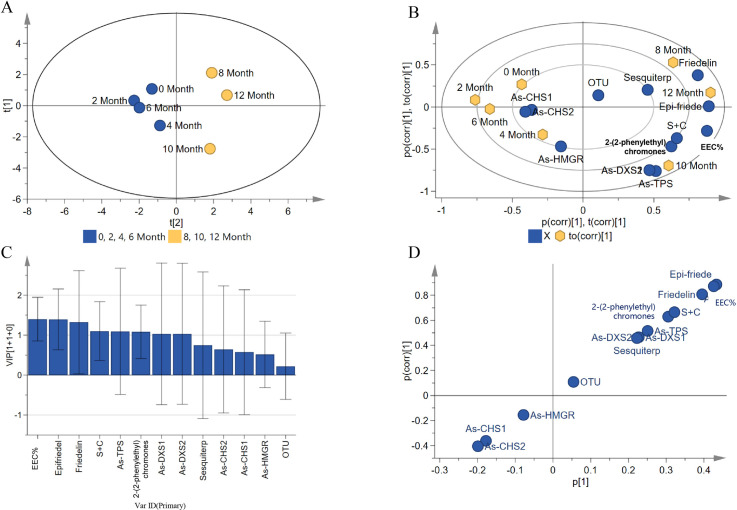
Multivariate statistical analysis of key indicators of agarwood and leaves of *A. sinensis* induced by FAA13. (A) OPLS-DA plot, (B) biplot, (C) VIP plot, and (D) S-plot plot.

Correlation analysis was conducted to investigate the dynamic changes of artificial agarwood and leaves induced by FAA13 over time. Multivariate statistical analysis was performed on key indicators, including expression of related genes, relative abundance of sesquiterpenes, relative abundance of 2-(2-phenylethyl) chromones, sum of relative abundances of sesquiterpenes and 2-(2-phenylethyl) chromones, alcohol-soluble extractive content, endophytic fungal abundance based on OTU of agarwood, and epi-friedelinol and friedelin content in leaves. Samples in PCA were divided into two groups based on induction time (Fig 10A): the first group was from 0–6 months, and the second group was from 8–12 months. The indicators that contributed the most to the grouping were identified using OPLS-DA Biplot (Fig 10B). The indicators *As*-*CHS1*, *As*-*CHS2*, and *As-HMGR* were clustered with 0–6 months, while indicators such as *As*-*DXS1*, *As*-*DXS2*, *As*-*TPS*, EEC%, epi-friedelinol, and friedelin were clustered with 8–12 months. The VIP method (VIP > 1.3) was used to screen out differential indicators contributing to the OPLS-DA grouping. As can be seen in Fig 10C, EEC%, epi-friedelinol, and friedelin were identified as the main differences between the two stages. In the S-plot diagram (Fig 10D), the indicator farthest from the origin was epi-friedelinol (the main difference indicator), followed by EEC% and friedelin. Overall, EEC%, epi-friedelinol, and friedelin were the main differentiating indicators between the induction groups 0–6 months and 8–12 months. These indicators were further quantitatively analyzed.

**Fig 11:**
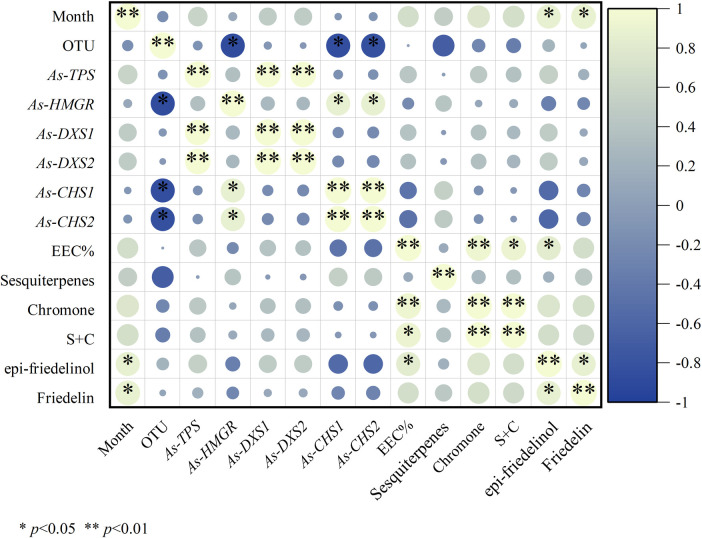
Bubble chart showing correlations of key indicators of agarwood and leaves of *A. sinensis* induced by FAA13 (S + C: sum of relative abundances of sesquiterpenes and 2-(2-phenylethyl) chromones).

Specifically, we examined the correlation between the abundance of endophytic fungi, the expression levels of candidate genes, EEC%, the relative abundance of sesquiterpenes and 2-(2-phenylethyl) chromones (S + C) in agarwood, and the content of epi-friedelinol and friedelin in leaves of *A. sinensis*(during FAA13-induced formation of agarwood (Fig 11).

There was a significant negative correlation between the relative abundance of agarwood endophytes induced by FAA13 and the expression levels of *CHS* genes. The expression level of the *TPS* gene was significantly correlated with the expression levels of the *DXS* gene. The expression levels  of the *HMGR* gene were significantly correlated with those of the *CHS* gene. EEC% showed a very significant correlation with the relative content of 2-(2-phenylethyl) chromones, and presented a significant correlation with the content of S + C and epi-friedelinol. Further, epi-friedelinol correlated significantly with friedelin.

### Analysis of key indicators in the agarwood and leaves of *A. sinensis* induced by FAA2

**Fig 12:**
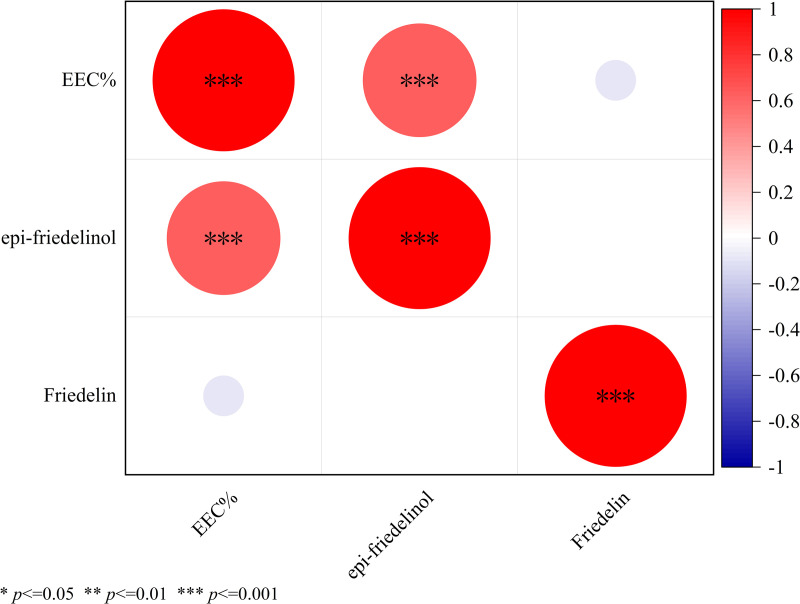
Bubble chart showing correlations of key indicators of agarwood and leaves of *A. sinensis* induced by FAA2.

In this study, we also analyzed the agarwood and leaves of another integrated induction method (FAA2) using a consistent analytical protocol. We used the same method to determine three key indicators in FAA2-induced agarwood and leaves, including EEC% in agarwood (Fig 8B) and the content of epi-friedelino and friedelin in leaves (Fig 7C). Notably, EEC% showed a very significant correlation with epi-friedelinol (Fig 12). Further, there was also a very significant correlation between epi-friedelinol and friedelin.

## Discussion

The integrated method of artificial induction can simulate the complex biological process of natural agarwood formation, and the agarwood it produces is closer to natural agarwood. The EEC% of agarwood is generally regarded as an important indicator for evaluating the quality of agarwood. This study confirmed that the artificial agarwood induced by FAA13 had high quality, and this induction method was closer to the natural formation process of agarwood. The EEC% of the FAA13 method ranges from 5.5% to 38.6%, which is higher than that obtained from other comprehensive stimulation methods, such as the combination of 1% formic acid with *Fusarium* sp. A2 (3.6%−15.0%) and the combination of 1% formic acid with *Xylaria sp.* A3 (11.7%−18.3%) [18,27]. Based on the analysis of key indicators of agarwood and leaves of *A. sinensis* induced by FAA13, we found that the samples were naturally clustered into two groups in the PCA (Fig 10A), which is consistent with grouping based on induction duration. In nature, plants may experience different types of stress from the external environment, broadly classified as biotic and abiotic stress [36]. On this basis, we speculate that the induction of *A. sinensis* by FAA13 occurs in two stages. According to PCA results, stage 1 was 0–6 months of FAA13 induction, and stage 2 was 8–12 months. During the one-year induction by FAA13, stage 1 mainly involves the acute stress response of plants dominated by abiotic stress. In contrast, stage 2 is characterized by the chronic response of plants to long-term stimuli dominated by biotic stress. Formic acid is the dominant contributor to abiotic stress throughout the process, while *B. rhodina* A13 can be considered the dominant contributor to biotic stress.

In stage 1, the leaves of *A. sinensis* fell off, indicating that the plants exhibited a stress response. The fungal diversity and relative abundance increased (Fig 2A, D), and the endophyte community changed from *Ascomycota* to *Basidiomycota.* This result indicates that the plant’s resistance to damage decreased while the endophytic fungi proliferated. 2-(2-phenylethyl) chromones, sesquiterpenes, and EEC% were not detected in 0-month samples but were significantly elevated in 2–6 month samples (Figs 6B, C and 8A). The expression levels of 2-(2-phenylethyl) chromones synthesis-related genes *As*-*CHS1* and *As*-*CHS2* are the most significantly increased in expression in this stage [23,27]. Consistent with the abiotic induction under salt stress, the induction process of the integrated induction method was verified. The stress effect of the early stage was mainly abiotic stress [23]. *As*-*CHS1* and *As*-*CHS2* represent the III polyketide synthase (PKSs) proteins, which can catalyze naringenin chalcone, the important intermediate for the biosynthesis of flavonoids [37]. The PKSs catalyze iterative decarboxylative condensations of malonyl unit with a CoA-linked starter molecule to produce structurally diverse, pharmaceutically important plant secondary metabolites [38]. *As-HMGR* is the key rate-limiting enzyme in the mevalonate (MVA) pathway [39]. The content of sesquiterpenoids was significantly increased at the 6th month, while the expression of *As-HMGR* was significantly increased, while other sesquiterpenoid synthesis-related genes were not significantly increased, and the synthesis of sesquiterpenoids at this stage was mainly related to the MVA pathway. In addition, as the expression levels of *As*-*CHS1*, *As*-*CHS2*, and *As-HMGR* genes increased, fungal diversity and relative abundance decreased sharply and were negatively correlated with these gene expression levels (Fig 11). The result provides evidence that *A. sinensis* can be induced by FAA13 to express genes related to 2-(2-phenylethyl) chromones and sesquiterpene synthesis, increasing EEC%. However, in the early stage of induction, the expression of 2-(2-phenylethyl) chromones-related genes is dominant, and therefore, the chemical compositions of artificial agarwood in this stage consist primarily of 2-(2-phenylethyl) chromones.

In stage 2, as the plant gradually enters the adaptive stage, *A. sinensis* grows new leaves, and the diversity and relative abundance of endophytic fungi gradually recover. Interestingly, the dominant endophyte community gradually shifted from *Ascomycota* to *Basidiomycota* in a similar fashion as stage 1 (Fig 2A). The expression levels of terpenoid synthesis-related genes *As-TPS*, *As-DXS1*, and *As-DXS2* are the most significantly increased in expression in this stage. The significantly increased expression of *As-TPS*, *As-DXS1*, and *As-DXS2* was opposite to the decreased expression of *As-CHS1*, *As-CHS2*, and *As-HMGR* genes. *As-DXS1* and *As-DXS2* are the key rate-limiting enzymes in the methylerythritol phosphate (MEP) pathway [24]. *As-TPS* is a key enzyme for the biosynthesis of sesquiterpene compounds and is important for agarwood formation in *A. sinensis* [40]. In the adaptation phase, the synthesis of sesquiterpenoids in *A. sinensis* is mainly related to the expression of MEP pathway-related genes and *As-TPS*. Gradual accumulation of secondary metabolites such as 2-(2-phenylethyl) chromones, sesquiterpenes, and EEC% was also observed, and the overall levels were higher than stage 1 (Figs 6B, C and 8A). This result suggests that the response time of sesquiterpenoid synthesis genes is slightly longer than that of 2-(2-phenylethyl) chromones synthesis genes after FAA13 induction. In contrast to stage 1, in stage 2, 2-(2-phenylethyl) chromones and sesquiterpenoid-related genes are positively correlated with fungal diversity and relative abundance. This indicates that plants and endophytes reach a balanced state of coexistence at this stage. This finding is consistent with previous research, which reported that *Ascomycota* dominates agarwood’s fungal community, and terpenoids appear to be closely related to fungal diversity, the primary determinant of agarwood characteristics [28,41].

In addition, we found that FAA13-induced volatile metabolites in the leaves were dominated by epi-friedelinol and friedelin (Fig 7A), with total relative contents ranging from 36.7% to 54.9%. Among them, epi-friedelinol is the derivative of hydroxylation of the carbonyl group by redox reaction at the C3 position of the carbon skeleton of friedelin. Epi-friedelinol and friedelin belong to corky alkane-type pentacyclic triterpenoid compounds, which are plant secondary metabolites widely distributed in nature, and they have anti-tumor, antioxidant, anti-inflammatory and neuroprotective bioactivities [42,43], which may be closely related to anti-inflammatory, analgesic and other activities of the leaves of *A. sinensis* [44]. There were two phases of FAA13-induced changes in the total synthesis of epi-friedelinol and friedelin in leaves, which were consistent with the two stages of artificially induced incense formation, with stage 1 being the pre-induction period (2–6 months) when the total content was lower than 0 months. The synthesis of epi-friedelinol and friedelin was lower than that of the catabolism in this stage. Stage 2 was the late stage of induction (8–12 months) with total content higher than 0 months, in which the synthesis of epi-friedelinol and friedelin was higher than the amount of catabolism. The leaves may respond to FAA13-induced stress through the synthesis of terpenoids and thus enhance the antimicrobial capacity of the organism, following the related reports that terpenoids can enhance the plant’s resistance to diseases and fungi [45]. Similar results were found in our further studies in FAA2-induced artificial agarwood. It is worth mentioning that we found that the content of EEC% of artificial agarwood induced by FAA13 and FAA2 was positively correlated with the content of epi-friedelinol significantly (Figs 11 and 12). The content of epi-friedelinol in the leaves showed the same trend as the content of EEC%, an important indicator of agarwood quality. In the two integrated induction methods of agarwood formation in this study, we found that the content of EEC% in agarwood stabilized and was at a high level in stage 2 (8–12 months). At the same time, friedelin rose rapidly from a plateau or after a slight decline, and epi-friedelinol continued to rise (Fig 7B, C). Therefore, we believe that during the production of agarwood by the integrated induction method, the appropriate harvesting period for agarwood should be determined by collecting leaves in stage 2 (8 months later) without damaging the tree and assessing whether friedelin enters a rapid rise from the plateau stage by rapid determination of epi- friedlinol and friedelin content. This non-destructive monitoring approach can prevent waste caused by tree felling.

## Conclusions

This work analyzed the changes in endophytic fungi and related gene expression, as well as EEC% and secondary metabolic spectrum in artificial agarwood induced by FAA13, and then determined their correlation. The findings revealed that the artificial agarwood induced by FAA13 was of high quality, and the FAA13 induction is a comprehensive and high-efficiency method for inducing agarwood. The induction process of FAA13 occurred in two stages. Stage 1 involved an acute response of plants primarily due to abiotic stress, while stage 2 involved a chronic response to long-term stimuli mainly caused by biotic stress. Eventually, plants and endophytes reached an equilibrium state where they coexisted. We also found that epi-friedelinol and friedelin were predominant among the volatile secondary metabolites of *A. sinensis* leaves. They may be important active substances in *A. sinensis* leaves. Among them, the contents of epi-friedelinol and friedelin in leaves can be used as an indicator to determine the harvesting period of agarwood during the induction process of the integrated method. The appropriate harvesting period for agarwood should be determined by collecting leaves in stage 2 (8 months later) assessing whether friedelin enters a rapid rise from the plateau stage by rapidly determining epi- friedlinol and friedelin content.

## Supporting information

S1 File Original data. The dataset includes all the original plotting data of this study, as well as qualitative and quantitative data of secondary metabolites.
(XLSX)
